# MacKillop Family Services’ Family Preservation and Reunification Response for Vulnerable Families—Protocol for an Effectiveness-Implementation Study

**DOI:** 10.3390/ijerph181910279

**Published:** 2021-09-29

**Authors:** Heather Morris, Melissa Savaglio, Nick Halfpenny, Renee O’Donnell, Alesia Pileggi, Andrea Dunbar, Robyn Miller, Helen Skouteris

**Affiliations:** 1 Health and Social Care Unit, School of Public Health and Preventive Medicine, Monash University, Melbourne 3004, Australia; melissa.savaglio@monash.edu (M.S.); renee.odonnell@monash.edu (R.O.); helen.skouteris@monash.edu (H.S.); 2 MacKillop Family Services, Melbourne 3205, Australia; nick.halfpenny@mackillop.org.au (N.H.); Alesia.Allford@mackillop.org.au (A.P.); Andrea.Dunbar@mackillop.org.au (A.D.); Robyn.Miller@mackillop.org.au (R.M.)

**Keywords:** family services, child protection, protocol

## Abstract

International evidence supports the effect of intensive family preservation and reunification services in preventing children’s placement in out-of-home care (OOHC). Evidence within Australia is scarce. This protocol paper describes a hybrid effectiveness-implementation evaluation of the Victorian Family Preservation and Reunification (FPR) Response implemented by MacKillop Family Services. Participants include families engaged in the program and staff involved in program delivery. A pre-post study design will be used to assess the effectiveness of the FPR in improving family outcomes from intake to closure, including: (i) parenting knowledge, skills, and capability; (ii) family safety and home environment; (iii) child development, adolescent behaviour, education attendance and attachment; (iv) connection to services; and (v) prevention of children from entering or re-entering OOHC. Interviews and focus groups will be conducted with staff to evaluate the program’s fidelity, reach, feasibility, acceptability, and enablers and barriers to implementation. Quantitative data will be analysed using descriptive statistics and a series of paired-samples t-tests and F tests to examine changes in outcomes over time; thematic analysis will be used for qualitative data. If the FPR can yield significant improvements in families’ outcomes, this would provide strong support for its scale-up across Australia, to better support vulnerable families.

## 1. Introduction

Intensive family preservation and reunification services are designed to keep children safe in the care of their parents and prevent child removal or subsequent placement into out-of-home care (OOHC; alternative accommodation when children cannot live at home safely [1]). They are effective in preventing children from entering OOHC 24 months post program engagement [2,3,4]. The effect of these programs is moderated by the sex and age of the child, parent age, the number of children and risk factors in the family, and practitioner caseload; greater effects (i.e., less OOHC placements) have been observed among families with boys and older parental age, whereas families with older and a greater number of children, single-parents, and higher caseloads were associated with reduced effects (i.e., more OOHC placements [5]). These programs have yielded additional positive outcomes for families, including reduced risk of maltreatment (particularly for high-risk families, [4]), and greater family functioning [5]. Finally, Tambling and Johnson (2020) concluded that increased provider-family contact (i.e., greater intensity of service) is associated with improved outcomes for families [6]. Despite these positive findings, additional primary research is required to establish a rigorous evidence base for intensive family preservation and reunification services, particularly in Australia where the literature is not well developed. Further, there is little known about how these programs work, the impact of parental adverse childhood experiences on current abuse and neglect [7] or the impact that person, social or environmental contexts have on the implementation of these programs [8]. These gaps in our understanding are a compelling rationale for the current study. This paper presents the study protocol of a mixed-methods evaluation of an intensive preservation and reunification program.

### 1.1. A Family Preservation and Reunification Services Program within One State of Australia

The Children Youth and Families Act 2005 gives a legislative basis for a system of services targeting vulnerable families [9]. In Victoria, it is the Department of Family, Fairness and Housing (DFFH) that runs child protection and investigates and manages the care of children with substantiated cases of abuse or neglect. In 2016, the Victorian government sought to review and reconstruct the service delivery system that engages children, youth and families following two royal commissions into: (1) child sexual abuse [10]; and (2) family violence [11]. The Roadmap for Reform: Strong Families, Safe Children [12] and the Roadmap to Recovery [13] are the blueprints used to restructure the system. The Victorian Family Preservation and Reunification Response (hereafter the Response, or FPR) is part of this restructuring to strengthen early intervention for disadvantaged families across the state and prevent/reduce OOHC placements [14].

In June 2020, a tender was put out requesting submissions from suitable community service organisations across Victoria who deliver family services to vulnerable families. Seventeen tenders were released to deliver the program across sites in both urban and regional settings. The goal of the Response was stated as being “strong families-with children who are safe, healthy, resilient and thriving; and parents who are supported to create a safe and nurturing home environment” [15]. The program seeks to provide intensive support to families where the children are at significant risk of being removed and placed in OOHC, or to enable the reunification of removed children in a timely manner. Referrals into the program are made via Child Protection, yet participation is voluntary. The FPR model is premised on the assumption that early, targeted and intensive support will permanently change the trajectory of vulnerable families away from the child protection (CP) system and towards the self-management of needs. OOHC placement often has adverse impacts on the child and family, and is extremely costly [16]. Hence, from a social, emotional and economic perspective, it is much better if families are supported and strengthened to ensure children can stay at home, safely. Table S1 outlines the government-led, individual core components of the Victorian FPR Response. Many are unique compared to other intensive family services by, for example, targeting adolescents, having a focus on implementation science (i.e., exploring *how* the program is being delivered), significant brokerage (i.e., funding to be used to purchase necessities for the family, such as groceries, housing appliances, contribute to rent, medication etc) and a new role within child protection called a Navigator. The Navigator is a government position that has been established within child protection to ensure that coordinated, targeted, appropriate, and culturally safe referrals are made into the FPR program. Rather than being prescriptive in program design, the Victorian government allowed all community service organisations to propose their own version of the FPR Response (within some broad parameters), with the view that consistency will be embedded through the government’s delivery of training and coaching to common practice elements (i.e., core components of program delivery) [17]. MacKillop Family Services was awarded nine areas of Victoria (six urban and three regional) to deliver the program.

### 1.2. The MacKillop Family Services FPR Program

MacKillop Family Services is a community service organisation that is committed to providing early intervention and family support services to disadvantaged families. Specifically, MacKillop strives to empower communities and families so that children can enjoy their childhood in a safe and loving home, can be healthy, thrive, and can develop to their potential. Key areas of intervention include operating foster care, residential care homes, homelessness support services, a suite of family service programs, specialist education services, and disability support and coordination services. In doing so, Mackillop supports children and young people to return to family, transition to independent living, heal from trauma, and to reconnect with learning and education. The FPR program aligns with MacKillop’s aim to provide early intervention for our most disadvantaged and vulnerable children, young people, and families. It addresses a key gap in the community—the lack of intensive family preservation and reunification services for high-risk and complex families. Therefore, the FPR program presents a significant opportunity to permanently change the trajectory of vulnerable youth out of the Child Protection system and OOHC, and capacity to breakdown the intergenerational cycles of maltreatment, OOHC placement, and disadvantage [15]. This protocol is necessary to outline and justify the program components, research processes, and measures being used in the evaluation.

The MacKillop FPR Response has eight core components in addition to the government’s key program guidelines. These include: (1) partnerships with other agencies to build expertise, local knowledge and networks; (2) embedding an independent evaluator; (3) utilising existing programs to incorporate into the Response; (4) extensive onboarding and training for staff; (5) being data-driven through assessment use to drive best practice; (6) a communities of practice for practitioners and team leaders; (7) organisational governance meetings; (8) suitable human resources to deliver the program and additional resources that enable program delivery with fidelity (i.e., as intended). More information about these components can be found in Table S2.

Families enter the program via the CP navigator. The navigator supports child protection practitioners to identify, engage, and connect the most appropriate families into the FPR program. Known as a ‘connection’ (i.e., referral or handover), the child protection practitioner, the Navigator, and MacKillop team leader discuss the family members, their circumstances, and past-history, prior to MacKillop accepting the family into the program. In alignment with collaborative practice and the two mandated Information sharing schemes (Child Information Sharing Scheme and Family Violence Information Sharing Schemes) [18,19], CP share all information relevant to the health and safety of the family and their workers. This includes mandated policy documents such as the Multi-Agency Risk Assessment and Management (MARAM) family violence screening and cultural support plans for Aboriginal and Torres Strait Islander children, as well as other known reports, such as mental health or disability assessments. Upon acceptance, MacKillop make contact within 48 hours and visits the family with CP within seven business days. These timeframes were prescribed by the Department of Family, Fairness and Housing. A thorough assessment of child and family outcomes is undertaken, and a ‘child and family action plan’ (i.e., care plan) is completed within 3 weeks that comprises the family’s goals, which are informed by those of CP. The plan is a living document that is revisited regularly to monitor progress and add or revise goals. CP workers are kept abreast of the family’s progress through attendance at care team meetings that are chaired by MacKillop Family Services workers.

The MacKillop key worker/practitioner engages with the family intensively, defined as a home visit for at least one hour, three times a week, or less visits and regular phone contact plus other activities as required. The timing of these visits is flexible to accommodate the needs of families. Funding is provided to allow for 200 hours of work with an additional 40 hours of step-down towards closure. It is expected that families will engage with the program for approximately six months, but this could last for up to a year. The most practical caseload per full time practitioner is four families, however workers may hold up to six families, albeit at a decreased intensity and with less fidelity to the model. In general, workers focus on reducing stressors and barriers to health and family functioning, such as housing, and offer referrals to specialist services if needed. Workers also focus on the interactions within the family and strengthen core life skills.

For families who are subject to an unborn report (reported to CP while pregnant) or with children aged 0–9, workers use evidence informed methods that focus on parenting skills (i.e., sleep settling, developing routines, responding to cues, use of consequences etc), parent–child attachment, and capacity to effectively respond during a crisis, such as those from the newborn behavioural observation method [20] or circle of security program [21]. These methods support families to meet their goals and mitigate risk. Specifically, the brokerage is a crucial component of the program that helps to fund items that meet families’ immediate or essential needs (i.e., groceries, rent), or can support families to achieve their goals (i.e., fund mental health or substance use counselling). As participation is voluntary and no specific incentives are provided, the brokerage may often be viewed by families as an incentive to engage in the program.

The program is also designed to support families to link into the community and its services to build independence, self-sufficiency, and a social network. Multi-disciplinary teams forged through partnerships with other agencies enhances the direct engagement of families with experts in parenting, drug and alcohol services, as well as regional care. Once families are better placed to meet the challenges they face and preferably with achieved goals, MacKillop will close the connection, but not before they provide the families with referrals to other programs or services if needed. For some families with young children, and where agreed to by the CP Navigator, an additional episode of care (240 h) will be provided to ensure that the family’s capacity is sufficient to keep children safe and thrive.

Families in metropolitan areas with young people aged 10–17 years engage with the evidence based Multi-Systemic Therapy Psychiatric (MST-Psych) program [22,23] where they see a trained MST-Psych MacKillop worker (termed family therapist) for at least one hour, three times a week. Full time family therapists are expected to hold up to four families and be available after hours where necessary. Family therapists adopt a systems approach by working closely with the child as well as the systems in which they live, e.g., caregiver, broader family members, home environment, school, peers, and community. They implement a range of evidence-based interventions, such as behavioural therapy, parent management training, cognitive behavioural therapy, and family therapy to meet the treatment goals within each system before closure. The regional MacKillop FPR programs also work with families of this age group, however they do not deliver an approved MST-Psych program, rather, they offer a service informed by this expertise.

Due to the lack of Australian evidence regarding the impact of intensive family preservation and reunification programs, a comprehensive mixed-methods evaluation of the MacKillop Family Services FPR program is being undertaken. In order to determine the effectiveness of the program, outcomes including parental knowledge, parenting skills, and capability; child development, adolescent behaviour and education attendance; connection to and use of family services; and protection from child abuse and neglect, were chosen in consultation with key stakeholders. These outcomes are associated with healthy, resilient and thriving children, and the work of family preservation and reunification programs [2,5]. Familial complexity, OOHC experience, and trauma, are risk factors for child abuse and neglect, which is under measured in current preservation programs [7]. These are being assessed to understand the factors that support program delivery. In addition to the assessment of effectiveness in relation to child, parent and family outcomes, the implementation of the FPR program is also being evaluated to inform future scale up [3,8].

### 1.3. Aim and Research Objectives

The overall aim of this paper is to outline MacKillop Family Services’ delivery of the FPR across nine sites and provide the research protocol for the three-year study period (October 2020–October 2023). This evaluation focuses on both parent/caregiver (hereafter parents) and child outcomes in relation to the effectiveness of FPR, and also seeks to evaluate the barriers and enablers, feasibility, fidelity and reach of the FPR program during the delivery and implementation phase.

Research objectives:

1. To use reliable and valid measures to assess the effectiveness of the FPR through changes in the following parent and child outcomes: (i) parenting knowledge, skills, and capability over time; (ii) family safety and home environment; (iii) child development, adolescent behaviour, education attendance and parent/child attachment; (iv) connection of parents to services; and (v) prevention of children from entering or re-entering the OOHC system over a long-term period (12 months).
2. To examine the implementation of the FPR by investigating the following outcomes: (i) the acceptability, appropriateness and feasibility of the program from the perspective of parents and staff; (ii) determine if the model can be implemented with fidelity (i.e., delivered as intended); (iii) evaluate the readiness of families to engage with the program, including reach; and (iv) examine the process, barriers and enablers of program delivery.

In addition to these research objectives, parental history of adverse childhood experiences and OOHC participation are being examined as risk factors contributing to current child abuse and neglect in response to recent gaps in the literature (Landers et al., 2018).

## 2. Materials and Methods

This paper was written using the Standard Protocol Items: Recommendations for Intervention Trials (SPRIRT) framework (2013) for writing protocol papers (see Table S3). The protocol was registered with Australian New Zealand Clinical Trials Registry (ANZCTR, 382402) and aligns with the World Health Organisation’s Trial Registration Data Set. Any changes made to the protocol will be communicated to the relevant parties (for example, investigators, ethics committee, trial registry and so on).

### 2.1. Ethics

Approval was obtained from the Monash University Human Research Ethics Committee to undertake the research study. MacKillop Family Services also reviewed and approved this research.

### 2.2. Study Design

This study aims to investigate the effectiveness and implementation of the FPR concurrently. To do so, this study adopts a mixed-methods study design involving a longitudinal single group pre-post study design combined with qualitative components. Given that participants are at high risk of child removal, it is not possible to include a control group, and a waitlist or similar group are also not viable. Other alternative study types are also not suited (for example, interrupted time series [24], because we cannot track participants for extended and lengthy periods of time). That is, given that the program’s duration is brief (200 h), highly intensive, and has a unique mix of outcome measures, any possibility for a matched control group regardless of prospective or retrospective data collection is prohibited. For these reasons, a quasi-experimental pre-post study design is being used to evaluate the effectiveness of MacKillop’s FPR program, acknowledging there are limitations to this approach. A multi-methods approach to data collection is being utilised, including focus groups, interviews, surveys, assessment questionnaires, and document analysis. Information from parents is being obtained only from those who provide consent. The study sites include all nine regions across Victoria, Australia, where MacKillop delivers the FPR.

### 2.3. Participants

There are two participant groups in this study: parents and MacKillop Family Services staff.

Parents: Parents either have children (0–17 years of age) who are vulnerable to removal from the home due to a child protection assessment of risk, have children already in OOHC, or are pregnant and have had ‘unborn’ reports to child protection. These parents may have multiple factors that are contributing to their risk profile including recent family violence, drug or alcohol misuse, fragile mental health, court orders and justice involvement among others. Parents can vary in age and may be adolescent parents or those who are currently living in a care arrangement (foster, kinship, or residential OOHC). Sample size calculations have been conducted using G*Power based on the NCFAS as this measure goes across most efficacy outcomes. For an analysis of variance test with the alpha set at 0.005, correlations between repeated measures set at 0.50 and power of 0.95, a total sample of 98 families will be required. To allow for a 25% rate of non-completion, at least 123 families will be recruited. During the study period, it is expected that up to 1100 families will engage in MacKillop’s FPR program. Based on similar research, we expect a high number of families to consent to participate in the study [25], therefore, it is expected that this sample size requirement will be met by the end of the study period.

MacKillop Family Services Staff: MacKillop directors, managers, team leaders and practitioners involved in the delivery of the FPR program are eligible to participate in interviews or focus groups about their experiences of implementing the program. Only staff who consent will take part in the research evaluation.

### 2.4. Recruitment and Informed Consent

Parents: All parents participating in the program are invited to participate in the current study through their key worker, however refusal to be in the study does not preclude them from receiving the FPR program. Due to the complexity of families and the need to have a trusting relationship established with the key worker, consent is likely to be obtained between the second and fourth months of program participation based on worker discretion. A flyer, written at a grade 5 literacy level and covering the most salient points of the study, is provided to all parents as they consider participating; declining participation has no effect on service delivery. After reading the flyer, an explanatory statement and consent form is provided (available on request), that requests approval for the receipt of the parent’s assessments and limited non-identifiable demographic data to be shared with the evaluation research team. For parents with children who are not adults, their legal guardian provides consent and the parent also needs to give assent. A separate explanatory statement and consent form have been developed for any parents who are interested in being interviewed during or post program participation.

MacKillop Family Services Staff: An explanatory statement and consent form is provided to all staff prior to participating in research activities like a focus group or interview. Choosing not to participate does not impact their current or future employment with MacKillop Family Services or their relationship with Monash University. Each of the nine teams comprises a team leader and approximately five key workers/practitioners. It is expected that the majority of staff will participate (*n* = 50 at minimum) as focus groups/interviews are being conducted during scheduled team meetings. Nonetheless, ongoing recruitment of staff will occur concurrently with data analysis so that saturation of themes can be assessed.

## 3. Data Collection

**Figure 1:**
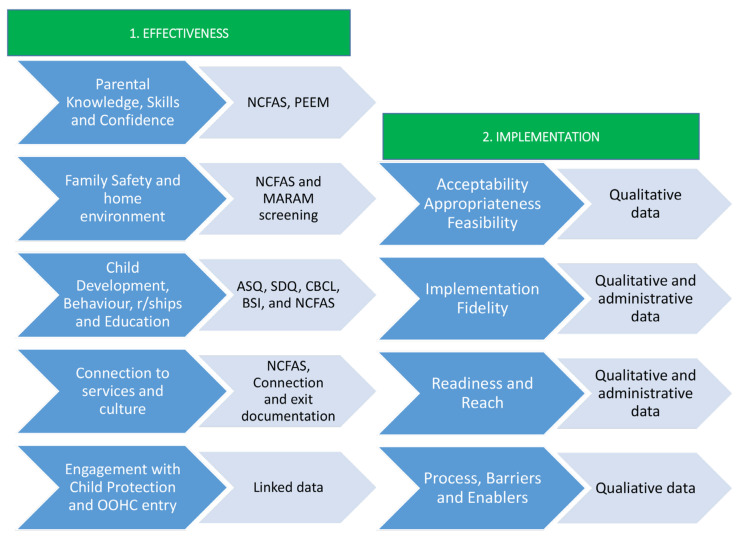
Outcomes and measures to evaluate the effectiveness and implementation of FPR. Note. NCFAS = North Carolina Family Assessment Scale; PEEM = Parent Efficacy and Empowerment Measure; MARAM = Family Violence Multi-Agency Risk Assessment and Management Framework; ASQ = Ages and Stages Questionnaire; SDQ = Strengths and Difficulties Questionnaire; CBCL = Child Behaviour Check List; BSI = Brief Symptom Inventory.

Figure 1 outlines the objectives, outcomes, and data being collected in the study, which is described in more detail below. While Figure 1 provides a snapshot of the outcomes and data, Table 1 offers specific details of all the data to be collected to answer the research objectives. All of the following assessments and administrative data are completed as part of standard practice of the MacKillop Family Services’ FPR program. Therefore, whilst data are collected for all families who engage in the FPR, only those who provide consent will have their data shared with the research team and included in the evaluation.

### 3.1. Outcome Measures for Objective 1–Effectiveness

#### 3.1.1. Parent and Family Focused Quantitative Measures

North Carolina Family Assessment Scale–for General Services and Reunification (NCFAS-GR) [26]:

The NCFAS-GR assesses change over time on ten family functioning domains that cover topics like safety, wellbeing, environment, and social life. The scale is completed by the FPR worker who accumulates knowledge about the family and then completes it in one sitting. Domain scores are measured using a six-point Likert scale ranging from “Serious Problem/Weakness = −3” to “Clear Strength = 2”. There is the option to provide a short comment to the score. The scores strongly guide the planning and practice, while also informing the child and family action plans and family goals. The NCFAS-GR is completed at the start of the program, after approximately 3 months or the middle, and upon closure. The NCFAS-GR has high internal consistency (*a* = 0.79–0.91) when implemented with families involved with Child Protection [27].

As per Figure 1, the NCFAS is being used to measure four of the five effectiveness objectives. The parental capability and self-sufficiency domains measure parent knowledge, parenting skills (i.e., sleep settling, developing routines, responding to cues, use of consequences etc) and parenting capability. The family safety and environment domains measure safety and security of the family’s home environment. Family interactions will be measured using the family interactions subscale of the NCFAS, which comprises eight items examining bonding with children, communication with children, expectations of children, relationship between parents/caregivers, support within the family, and family routines, rituals, and activities. The first three items provide an indicator of parent–child attachment, while the combination of all eight items yields an overall score of family interactions. The child wellbeing and family health domains examine the child’s health and development. In addition, the social life domain measures the child’s connection to education, as well as the parent’s connection to community services.

Parent Efficacy and Empowerment Measure (PEEM) [28]:

The PEEM is comprised of 20 positively framed statements about parenting that are rated by the parent on a scale from one to ten. The measure examines a parent’s feelings of control and confidence in parenting, especially when challenges present. Therefore, it is being used to determine the effectiveness of the FPR in improving parental confidence. The measure has been developed with an understanding of social work practice and information is available to support workers to use it appropriately, and it has high internal consistency (*a* = 0.92) [29]. Parents complete the PEEM during a session with their FPR worker, and it can be used as a tool to support disclosure and relationship building. The PEEM is completed at the start and upon closure by all families except for MST-Psych participants.

#### 3.1.2. Child Focused Quantitative Measures

Ages and Stages Questionnaire [30]: This screening tool, completed by the FPR worker, measures progress towards developmental milestones in children up to 60 months in age. All children up to five years of age are assessed using this measure. There are 21 measures based on age in months, each with items that are appropriate for the selected age, with high internal consistency (*a* = 0.86) [31]. As such, a different survey is used between baseline and closure depending on the child’s age. Scores indicate if the child needs referral to specialist services or extra monitoring. This measure is completed at the start of the program and upon conclusion.

Strengths and Difficulties Questionnaire (SDQ) [32]: The SDQ comprises 35 items about the psychosocial functioning and behaviour of the child, and hence responds to the FPR’s impact on child wellbeing. There are 25 statements rated as ‘not true’, ‘somewhat true’ or ‘certainly true’, five short answer questions and an additional five items, which rate the impact of the difficulties shown. This measure is used by parents about their children who are aged 6–9 in all metropolitan areas or for children aged 6–17 in regional areas as they are not receiving the MST-Psych program. Attempts are made to use the self-report youth measure for children over 10 years of age, however where this is not possible, the parent reports on the child’s behalf. Both versions have moderate to good internal consistency (*a* = 0.53–0.86) [33]. The SDQ is completed at the start and upon conclusion.

Child Behaviour Check List (CBCL) [34]: The MST-Psych program uses the Child Behaviour Checklist. This measure is completed by the parent and/or young person with the support of the FPR MST family therapist to outline problem behaviour in children and responds to the child behaviour objective. Respondents are invited to consider their own/their child’s behaviour in the last six months. There are eight individual syndrome scales with high internal consistency (a = 0.90–0.97) [35]. Two scales are derived by combining scores from some of the syndrome scales to determine internalising or externalising problems. Scores can be compared to a normative sample to determine the severity of the problem, which can be classified as normal, borderline or clinical. This checklist is completed at baseline and upon program closure.

Brief Symptom Inventory (BSI) [36]: The MST-Psych program also uses the BSI, which has 53 items that examine nine system areas of psychopathology, with each rated on a five-point Likert scale from 0 (not at all) to 4 (extremely). Respondents are invited to consider the intensity of their distress over the past 7 days, and the severity of the problems can be calculated. This measure is used to understand adolescent wellbeing and behaviour, with strong internal consistency (a = 0.93) [37]. The BSI is collected by the adolescent (aged 10–17 years) at baseline and upon program closure.

#### 3.1.3. Parent and Family Focused Qualitative Data

Parents will be given the opportunity to provide feedback about their experience of the FPR program in a semi-structured interview lasting between 30 and 45 minutes. Questions about skills and confidence, parent/child relationships, connection to services, wellbeing, safety and home environment will be asked only to those who choose to participate. The researcher will rely on the expertise of practitioners to engage parents who can speak to their progress. We acknowledge that this approach may lead to confirmation bias, however we feel this is ethically appropriate and is in agreement with MacKillop Family Services staff views. The key worker will provide an explanatory statement and consent form to be completed prior to the interview. It is not a prerequisite that they have signed the consent form for sharing their quantitative measures to complete the interview. The interviews will take place during one of their home visits and the key worker will be there to support the parent pre- and post-interview. Due to COVID-19 pandemic restrictions, geographical distance, and privacy/confidentiality, the interviews will occur over the key worker’s phone or via Zoom, depending on the preference of the parent.

#### 3.1.4. FPR Effectiveness in Reducing Reports to Child Protection and Subsequent Placement into OOHC

To understand if the MacKillop Family Services FPR has been successful at reducing family involvement with child protection and preventing children from entering/re-entering OOHC, three types of linked data from the government are being sourced, subject to approval: (1) reports made to child first, or other child protection organisations who use the Client Relationship Information System (CRIS) system, about FPR children and parents in the six months before participating in the FPR, the six months during and then six months after program closure; (2) number of times children have been in OOHC in the 12 months post FPR participation; (3) time to reunification after removal; or (4) time to removal/re-removal.

#### 3.1.5. Supporting Administrative Data for Effectiveness Evaluation

Extensive administrative data are being used to support the effectiveness evaluation of the FPR. The connection form (i.e., referral document) and its attachments (such as family violence screening tools, diagnoses of disabilities, prior OOHC history of parent and so on) provide detailed information to support our understanding of parental context, connection to services and culture, and existing safety concerns. The closure checklist, which is completed by the key worker, also provides information about the service and education connection and participation.

### 3.2. Outcome Measures for Objective 2–Implementation

Table 1 describes the implementation processes that MacKillop Family Services are evaluating, the data it is using, and how it is being sourced. Much of these data are qualitative and sourced directly from MacKillop Family Services staff. Being respectful of the workload and time pressures faced by the key workers, they are only being engaged every six months in a focus group during a regularly planned team meeting. This will ensure that their capacity to keep families as their focus is not compromised. Approximately five key workers from each of the nine teams will participate in the focus group, totalling a possible sample size of 45 participants. The nine team leaders are interviewed every fortnight for 30 min to understand the immediate pressures and issues that their teams are facing, while their managers are interviewed every quarter.

#### 3.2.1. Acceptability, Appropriateness and Feasibility

Acceptability, appropriateness and feasibility is being assessed using qualitative methods at both the parent and staff levels. While validated measures for these factors exist, including the Acceptability of Intervention Measure (AIM), Intervention Appropriateness Measure (IAM) and Feasibility of Intervention Measure (FIM) [38], the considerable number of measures for parents and the key workers being time poor, make their use infeasible despite their brevity. This decision was made in conjunction with MacKillop staff.

#### 3.2.2. Fidelity

Implementation fidelity is being measured, relating to the connection, assessments, information sharing, adherence and quality. Parent readiness and the program reach is also being examined. Much of the data are sourced through case notes, presence of certain documents in the case file and routine reporting.

#### 3.2.3. Readiness and Reach

Readiness is assessed through the connection form (i.e., referral form obtained from child protection), case notes and the first PEEM completed by parents at baseline. The PEEM offers a snapshot of parent reflexivity and understanding of their parenting practices. For example, if parents score themselves 9 or 10 for most questions, this suggests that they may not be ready to undertake work on their parenting skills. Program reach is determined by the number of clients who participated fully in comparison to the number who refused service.

#### 3.2.4. Process, Barriers and Enablers

The Consolidated Framework for Implementation Research [39] has five domains (Intervention, Outer Setting, Inner Setting, Individuals, and Process) to guide the analysis of the qualitative data as barriers and enablers are identified. These factors will be examined through the lens of the implementation cycle [8], which states that implementation takes between two to four years to do, each with stages. As such, some factors will not be relevant in the early stages compared to later stages. An examination of the process, barriers and enablers as they emerge is critical for MacKillop Family Services to actively respond and manage barriers as they arise.

#### 3.2.5. Parent Trauma History

Parental trauma history will be examined in two ways: (1) examination of connection documentation for personal OOHC experience; and (2) through the Adverse Childhood Experiences (ACEs) survey [40]. The connection documentation is the referral form which is provided by child protection to MacKillop Family Services before the family is accepted into the program. This form indicates numerous demographic characteristics regarding the parents, including whether they have had personal OOHC experience. The ACEs survey comprises seven categories of abuse or dysfunction, each with one or more questions; three categories are about childhood abuse that could be either psychological, physical or sexual; and four categories of household dysfunction including exposure to substance abuse, mental illness, violence towards a caregiver and criminal behaviour. Responses are scored as yes/no and adult respondents are asked to think about any time up until they were 18 years of age. The ACEs survey is completed on one occasion (towards the end of program engagement) with the FPR worker.

## 4. Analytical Considerations

The primary quantitative outcome of this study is to examine the degree to which there are improvements in psychosocial outcomes among families who engage in the program. All completed questionnaires will be de-identified by a specific data aggregation worker who will assign a unique ID code (e.g., FPR003) to each family so that their data remain anonymous to the research team. Descriptive statistics will be calculated based on administrative and demographic data sourced from the referral form. Main caregiver complexity will be determined using the ACEs survey and the number of ACEs will be calculated. A series of paired sample t-tests (repeated measures) will be used to compare participants’ mean differences on the measures at the beginning and end of the program. Analysis of Variance will be employed to review the number of reports before, during and after the program to determine any statistical differences. The number of care episodes and time to reunification will be analysed descriptively. All analyses will be performed using the Statistical Package for the Social Sciences (SPSS) software.

All qualitative data will be analysed using thematic analysis [41]. Data collection and analysis will occur concurrently so that data saturation can be adequately assessed. All audio recordings will be transcribed verbatim and systematically coded. Following in-depth review of the coded data, independent themes will be identified and developed based on recurrent content reflecting the different aspects of program implementation. The process of refining and reviewing themes will be an iterative process until themes are representative of the most pertinent and recurrent aspects of the data.

### 4.1. Data Management

All project materials, including data, are stored on a secure password-protected online database. Only the chief investigator from Monash University has access to the data set. All data will be deleted six years following the conclusion of the project. Any adverse events that occur as a result of this project will be reported to the Monash University Human Ethics Committee and managed accordingly. An independent audit will occur annually to ensure appropriate trial conduct is upheld and that the data collected and stored is accurate and confidential. Given this, a data monitoring committee is not necessary.

### 4.2. Dissemination of Findings

The findings will be disseminated to MacKillop Family Services via regular meetings and reports to the organisation throughout the three-year evaluation period. A summary of findings will be shared with participants. It is also intended that the findings of this hybrid effectiveness-implementation evaluation will be disseminated with the public via a series of publications in peer-reviewed journals. The research team from Monash University and key stakeholders involved in the delivery of the program from MacKillop Family Services will be included as authors on such publications, where relevant.

## 5. Discussion

This paper describes the protocol of a hybrid style, implementation and outcomes study using a pre-post design with selected follow-up data and qualitative data. The study will serve two key purposes. First, it will seek to demonstrate the extent to which the Response is effective in improving outcomes for families and children following the program. Whilst this is not a randomised controlled trial, the pre-post design will allow some conclusions to be made about the potential impact of the program on engaged families. In turn, this will develop the Response’s evidence base and provide new knowledge about the implementation of intensive family services more broadly. Second, the findings of this study, particularly the implementation findings, will help inform future iterations of the program. It is intended that key program components, processes, or operational issues may be adapted, refined, or remedied to improve implementation and uptake of the Response. Therefore, the evaluation will help ensure that the program is trialled, tested, and adapted accordingly to effectively meet the complex and unique needs of some of the most vulnerable families in the Victorian community.

## 6. Conclusions

The current study is necessary and critical for generating Australian evidence regarding the effectiveness of an intensive family service program that aims to preserve or reunify families through the provision of assertive outreach support. Importantly, if the program is found to be well-implemented and yields significant improvements in families’ outcomes from baseline to closure, then this would provide strong support for the scale-up and dissemination of the Response across Australia. In turn, this could begin to foster the intergenerational outcomes that many of the target families currently experience.

## Figures and Tables

**Table 1:** Effectiveness and implementation outcome measures.

Outcome	Individual Measures	How Measured	When Measured
	Program Effectiveness Outcomes		
Parental knowledge, skills, and confidence	Self-efficacy and empowerment Parenting capability and self-sufficiency	Connection form—parent strengths and capacity section; PEEM; NCFAS-parental capability and self-sufficiency subscales	NCFAS at baseline, mid and closure; PEEM at baseline and closure
Family safety and home environment	Recent family violence screens (MARAM) Child protection notifications OOHC entry/re-entry	Connection form NCFAS—family safety subscale, environment subscale MARAM—intermediate screen Linked data from the Victorian Government	NCFAS at baseline, mid and closure; MARAM at intake
Child development (early years) or behavior (adolescence), relationships and education	Looking at changes in child development over time Is there a good parent/child relationship What is the child’s connection to education	NCFAS—Child Wellbeing, Family health subscales, Family interactions; ASQ—Child development up to 60 months SDQ—Adolescent Behaviour (regional) CBCL—Adolescent Behaviour (metro) BSI—Adolescent Behaviour (metro) Connection form; Closure checklist; Closure goals; Client interviews and exit survey	NCFAS at baseline, mid and closure. Others at intake and closure
Connection to services and culture	Are Aboriginal and Torres Strait Islander families supported in their cultural connection.	Connection form; NCFAS—social life subscale; Closure checklist; Cultural support plan of Aboriginal and Torres Strait Islander children are provided; Families are connected to cultural communities; Staff have cultural training and access to a cultural advisor	NCFAS at baseline, mid and closure. Baseline for connection form; and closure for closure checklist
Engagement with Child Protection and OOHC Entry	How much do families engage with Child Protection? Child protection notifications Length of time between connection received and reunification Time to removal/re-removal	Linked data from the Victorian Government Case Notes; NCFAS—Ambivalence and readiness subscales; Connection form	NCFAS at baseline, mid and closure
**Implementation Outcomes**			
Acceptability Appropriateness Feasibility	Do clients think the program is acceptable? Is the FPR the best program for these clients? Can clients feasibly engage intensively with the program? Can FPR workers feasibly do the work in the time available?	Completion rates via internal reporting; Interviews with clients; Exit survey; Client interviews Staff focus groups; Team leader interview	Acceptability—At closure Appropriateness—At closure; during program delivery Feasibility—Mid program and Closure
Implementation Fidelity ◆Connection	FPR made connection within 2 working days; Met with CP within 1 week;	Case notes	At baseline
◆Assessments	Initial assessment completed in a week; Comprehensive in three weeks	Case notes	At baseline
◆Information Sharing	All appropriate forms and documents are supplied	Complete connection forms; MARAM screenings, and cultural support plans where relevant	At baseline
◆Adherence to the model	Intensive engagement (dose) FPR worker engaging in the first 2 working days of connection Joint visit with Child Protection in the first 7 days of connection	Program reporting and case notes about hours of engagement including the total number; program period; Dates of connections and visit	At baseline and closure
◆Quality	Program completed; Relationship is strong and established	Completed program as found in case notes; exit survey. Interviews with families, and staff.	At closure
Readiness and Reach	Is the client ready for change and willing to engage with the program? What is the proportion of clients who participated fully? How many clients refused or the service refused?	Connection form; Interviews with family; PEEM baseline survey; case notes Program documentation: Clients completed program/clients who closed early	At closure
Process, Barriers and Enablers	What are the barriers and enablers to the program’s delivery? CFIR domains: ◆Relative advantage ◆Adaptability ◆Complexity ◆Client needs and resources ◆Structural characteristics of the team ◆Communication ◆Goals and feedback ◆Learning climate ◆Knowledge and beliefs ◆Personal attributes ◆Intervention characteristics	Staff interviews and focus groups	Regular interviews with team leaders on a fortnightly basis; Staff focus groups every 6 months
**Understanding context**			
Parent context	Mental health; Family violence; Current AOD use; Court orders; Risks and needs	Connection form; ACEs survey	ACEs completed by FPR worker mid to late program

## Supplementary Materials

The following are available online at https://www.mdpi.com/article/10.3390/ijerph181910279/s1, Table S1: Core features of the FPR; Table S2: Key components of MacKillop’s FPR, Table S3: SPIRIT checklist.
