# Supplementary material for: MacKillop Family Services’ Family Preservation and Reunification Response for Vulnerable Families—Protocol for an Effectiveness-Implementation Study
Source: Int J Environ Res Public Health. 2021 Sep 29;18(19):10279. doi: 10.3390/ijerph181910279 (PMC8508066; doi:10.3390/ijerph181910279)
Supplement: Supplementary file 1 [file ijerph-18-10279-s001.zip › Table S1_FPR Components.pdf]

Table S1

Key features of the FPR program

FPR Component	Description
Child Protection Navigator	The Child Protection Navigator role is a new position specifically designed for the FPR program that has been established within Child Protection. This role seeks to ensure coordinated, targeted and culturally safe referrals are made into the FPR program. The navigator is responsible for supporting Child Protection practitioners to identify, engage, and connect the most appropriate families that meet the FPR eligibility criteria. The navigator will also coordinate case allocation conferences between the FPR practitioners and Child Protection following a referral.
Lead Practitioner	Each family involved in the program is allocated a key FPR practitioner. The practitioner is the main contact person for the family who is responsible for delivering intervention and support in a timely and rapid response. The FPR practitioners have a caseload of approximately four families each, and conduct regular home-visits, outreach support, and case management to meet each of families' unique needs. They use culturally safe, evidence informed, and trauma-informed strategies to support family engagement, trust, and to build the capacity of the families for reunification or preservation.
Relational Trauma-Informed Approach	The FPR program provides a culturally safe, trauma informed and inclusive response for all families. The relational trauma-informed approach aims to understand and respond to the specific needs of each family, by building trust, safety, and a positive therapeutic relationship with the family, modelling secure attachment, and seeking to interrupt the cycle of intergenerational trauma. For Aboriginal children and families, this approach will support cultural healing and recognise and understand the unique needs, preference, and history of Aboriginal children and families by adjusting the Response based on the individual needs of each family and community.
Evidence Informed Approach - The Common Elements	All families are provided evidence-informed support. FPR practitioners have undergone extensive training to deliver services to families using the evidence-informed practice elements (common elements). Such elements are outlined in the Practice Modules that have been specifically developed for the FPR program[1]. The extent to which these common elements are implemented with fidelity will be assessed to ensure that the FPR is being delivered as intended.
Co-designed and collaborative care plan	A co-designed care plan is devised in collaboration with Child Protection, the FPR practitioner and family involvement. Specifically, FPR practitioners use a strength-based approach that puts the child at the centre and recognises families are the experts in their experience. The practitioners seek to empower the voice of the family in decision-making about services and support, which informs the family's care plan. Throughout service delivery, children and families will be empowered from connection to closure to set, work towards, and achieve goals that they choose, guided by Child Protection.
Connected and Joint Practice Arrangements	A defining feature of this program model is that Child Protection and the FPR team are required to work collaboratively to achieve the family's goals outlined in the co-developed care plan. The FPR practitioners facilitate an FPR care team around the child and family that is focused on the achievement of such goals. The navigator is the gateway person that facilitates the FPR Response team and Child Protection to work in collaboration to deliver intensive and timely support.
Strong local area governance	The Response governance aims to build on and align to existing locally developed Child and Family Service Alliances across Victoria. The goal is to progressively increase membership to the Alliance from across the community, education, health and justice sectors. An FPR Operations Group will oversee the delivery of FPR, including progress towards outcomes.
Implementation Science focus with an Evaluation embedded	FPR will be part of a state-wide evaluation of the Response conducted by the Centre for Evaluation and Research, Department of Health and Human Services. A multi-phase monitoring and evaluation framework will be developed in collaboration with service providers. The evaluation framework will examine program implementation and key program outcomes to build the 'evidence-based' status of the program over time.

Note. Information adapted from https://providers.dhhs.vic.gov.au/victorian-and-aboriginal-family-preservation-and-reunification-response.

[1]The practice modules are not available online and can not be referenced as they are the propriety of the Victorian Government.
