# Supplementary material for: MacKillop Family Services’ Family Preservation and Reunification Response for Vulnerable Families—Protocol for an Effectiveness-Implementation Study
Source: Int J Environ Res Public Health. 2021 Sep 29;18(19):10279. doi: 10.3390/ijerph181910279 (PMC8508066; doi:10.3390/ijerph181910279)
Supplement: Supplementary file 1 [file ijerph-18-10279-s001.zip › Table S2_Components table_MacKillop.pdf]

Table S2. Key components of MacKillop's FPR

Element	Description	Rationale
Partnerships	There are several partnering organisations including the Queen Elizabeth Centre (QEC); Odyssey House; O'Connell Family Centre; Gateway Health; Mallee District Aboriginal Service (MDAS); Bendigo District Aboriginal Cooperative (BDAC); Catholic Care Sandhurst; and Victorian Aboriginal Child Care Agency (VACCA).	These organisations provide expert knowledge of their respective fields as well as local area expertise.
Researcher-in-residence	MacKillop Family Services embedded external researchers at the program's outset. They have implementation science expertise to independently investigate and iteratively develop the program, feedback data and evidence, and evaluate the program.	The researcher-in-residence model is a critical element to ensure the successful delivery of project outcomes. It will enable a rigorous understanding of 'how' the FPR program works, not just the 'if' it works, which is aligned with the Victorian government's purpose of the response.
Current Programs	The MacKillop Family Services FPR model offers MST Psych for children aged over 10 and has based its program for younger years on the Cradle to Kinder programs. These have an evidence base supporting their use.	MacKillop Family Services chose to implement the MST-Psych program because it aligns with the program's aims by providing assertive outreach and systemic support to families with young people at risk of OOHC placement due to significant co-occurring behavioural and mental health concerns. Additionally the program was pre-existing within MacKillop thus the infrastructure, connections and relationships were already in place quickly.
Training	MacKillop Family Services offers extensive training last from 2 hours, up to 3 days, including: organisation's processes (Welcome day, the Sanctuary model, mandatory competencies, policies and procedures); Reporting (Client Incident Management System (CRIS); Integrated Reports and Information System (IRIS); Family Journey); Response specific training (Assessments; MARAM framework; Newborn Behavioural Observation; Child Focused Family Centred Practice; Leadership (supervision training for team leaders); and cultural awareness.<br><br>Additional training is provided by the Victorian government about the common elements practice modules, the details of which are out of scope for this paper.	The training offered will provide practitioners and staff with the knowledge to broaden and hone their practice expertise to strengthen outcomes for families.

Table S2. Key components of MacKillop's FPR

Data driven through assessments	All assessments except for the ACE survey are tools regularly used to strengthen practice.	The assessments that were selected for inclusion in this study have a strong social work focus designed to improve practice, link data to service provision, inform the child and family action plan, track progress, screen for developmental concerns and strengthen the safety of families and staff. The data will be rapidly analysed and fed back into the system for review, consolidation and decision-making.
Communities of Practice	A community of practice was set up for all FPR team leaders to participate in	This initiative was an avenue for quick decision-making, a collaborative process for policy and document development, as well as a reflective exercise. Author AD was responsible for the initiation of the community of practice meetings and these meetings occur monthly.
Governance	Several meetings were established to ensure the FPR's delivery occurs smoothly, with decision making and problem solving a priority including: 1) a Steering Committee chaired by the CEO of MacKillop Family Services (author RM); 2) A monthly evaluation working group chaired by author NH, that is attended by team leaders, the director for policy and research, and the researcher in residence (Author HM); 3) a fortnightly meeting with senior leadership, managers and team leaders to ensure that messaging from the frontline workers reaches the highest level of decision makers quickly and efficiently; and 4) a monthly, local implementation team meeting.	The steering committee was designed to ensure a smooth transition of the program from concept into practice. In 2020 the FPR Steering Committee met monthly and included representatives from all partners, and this meeting is now biannual. The evaluation working group is essential for questions related to research, while the fortnightly meeting ensures that all levels of leadership are able to communicate and problem solve internal issues. A monthly local implementation team meeting also occurs with a government representative who supports the discussion of barriers and enablers beyond the internal organisational level.
Staff Resourcing and support	MacKillop Family Services provide in most cases, their family workers with a car that they can take home rather than having to book. They have the ability to work from home, all personal protective equipment for home visits is provided, a computer, regular debriefing, reflective practice and supervision fortnightly, clinical reflection and coaching to the common elements monthly.	Having a car that is allocated and available to take home rather than having to go into an office to collect it, saves time and resources and offers some work-life balance, as so does the ability to work from home to do administration tasks. Debriefing and supervision are tools supporting worker safety and support professional practice. Reflective practice, clinical reflection and coaching all offer professional support to reduce program drift and strengthen fidelity.
